# Dynamics of auditory working memory

**DOI:** 10.3389/fpsyg.2015.00613

**Published:** 2015-05-11

**Authors:** Jochen Kaiser

**Affiliations:** Institute of Medical Psychology, Goethe University, Frankfurt am Main, Germany

**Keywords:** review, event-related potentials, spectral activity, gamma, coupling, spatial processing, non-spatial processing

## Abstract

Working memory denotes the ability to retain stimuli in mind that are no longer physically present and to perform mental operations on them. Electro- and magnetoencephalography allow investigating the short-term maintenance of acoustic stimuli at a high temporal resolution. Studies investigating working memory for non-spatial and spatial auditory information have suggested differential roles of regions along the putative auditory ventral and dorsal streams, respectively, in the processing of the different sound properties. Analyses of event-related potentials have shown sustained, memory load-dependent deflections over the retention periods. The topography of these waves suggested an involvement of modality-specific sensory storage regions. Spectral analysis has yielded information about the temporal dynamics of auditory working memory processing of individual stimuli, showing activation peaks during the delay phase whose timing was related to task performance. Coherence at different frequencies was enhanced between frontal and sensory cortex. In summary, auditory working memory seems to rely on the dynamic interplay between frontal executive systems and sensory representation regions.

## Introduction

Working memory allows the temporary storage of relevant information and its task-dependent manipulation. It is involved in many higher cognitive functions and thus constitutes a fundamental function of our brain. While most previous research has focused on visual working memory (Drew et al., 2006; Luck and Vogel, 2013), less is known about the neural correlates of auditory working memory (AWM). This brief review summarizes some of the main findings on auditory short-term or working memory (both terms will be used interchangeably) studies in humans. The focus will be on the dynamics of working memory-related processes; therefore the review is limited to studies assessing non-invasive measures of neural activation with a high temporal resolution, i.e., electro- or magnetoencephalography (EEG and MEG). Most of this work has considered event-related potentials (ERPs), but some investigations have looked at spectral activity and at oscillatory coupling between cortical sources.

Evidence from both types of studies speaks against the existence of a single working memory store for auditory information. Instead, activation patterns vary with the type of memorized auditory information, suggesting that working memory involves the same systems that underlie perceptual processing. Sound feature-specific activation differences were particularly obvious for comparisons between sound identity and location, i.e., stimulus parameters that are processed in topographically distinct cortical regions (Rauschecker and Tian, 2000).

## Auditory Working Memory for Non-spatial Sound Features

The short-term retention of pitch elicits a load-dependent frontal negative wave. Using non-verbal, pure-tone stimuli to avoid phonological or semantic processing, memory load effects were tested by presenting either one 200-ms pure tone to both ears or two different stimuli to each ear (Guimond et al., 2011). A sustained anterior negative wave (SAN) during the 2-s delay interval showed higher amplitudes for two than one to-be-remembered stimulus. Control experiments confirmed the role of the SAN for short-term memory processing by excluding a mere sensory-driven response or internal rehearsal. Comparison with a visual short-term memory paradigm showed that the SAN during retention was specific to the auditory task (Lefebvre et al., 2013). A memory load-sensitive SAN was also observed during the retention of sounds differing in timbre instead of pitch (Nolden et al., 2013). The cortical generators of this wave were assessed with MEG. During AWM for tone sequences, source localization revealed memory load-dependent activations in bilateral superior temporal, superior parietal and frontal cortex (Grimault et al., 2009). A study involving the comparison of tone sequences of different lengths identified several brain areas whose activation correlated with the number of successfully memorized items (Grimault et al., 2014). These included bilateral superior/middle temporal cortex and several regions in bilateral frontal cortex. This source topography partly overlapped with fMRI results (Gaab et al., 2003; Koelsch et al., 2009) and suggested that the retention of simple acoustic features involves the sustained activation of sensory representations in addition to frontal executive regions.

The frontal negativity is a robust phenomenon that was also observed in ERP studies employing verbal sounds that may elicit semantic processing beyond low-level acoustic storage. A sustained frontal negative shift was larger for aurally than visually presented digits (Lang et al., 1992). Similarly, a memory load-dependent frontal negativity was larger for spoken than written syllables (Ruchkin et al., 1997), whereas visual stimuli gave rise to a posterior positivity. The role of the prefrontal cortex for AWM was further supported by a study in patients with frontal cortex lesions. They showed reduced activations both in auditory areas and prefrontal cortex and failed to attenuate their responses to distracting tones during the delay period of an AWM task (Chao and Knight, 1998).

While ERP investigations focus on time-locked broad-band activity, spectral analysis is typically performed on single-trial basis, maintaining activity that is not phase-locked to a defined event. Analyses of spectral activity in different frequency bands may inform about aspects of processing not captured by ERPs. For example, activity in the alpha band (8–12 Hz) has been related to active inhibition of interfering processing (Klimesch et al., 2007; Jensen and Mazaheri, 2010), and gamma activity (>30 Hz) has been linked to object representations, attention and memory (Kaiser and Lutzenberger, 2003; Jensen et al., 2007). Moreover, coherence or phase synchronization calculated on the basis of spectral signals provide information about cortico-cortical interactions.

Increases of spectral power and synchronization over frontal cortex characterized AWM for different types of non-spatial sounds. During the maintenance phase of an AWM task requiring the memorization of sound durations, we found increased gamma activity (70–80 Hz) over prefrontal cortex (Kaiser et al., 2007b). A similar result was obtained for artificial syllables varying in voice onset time and formant structure. Here gamma activity (65–70 Hz) was increased over left anterior temporal/inferior frontal cortex (Kaiser et al., 2003). Gamma coherence between the putative sensory representation regions and prefrontal cortex showed a sustained increase across the delay phase (Kaiser et al., 2005), possibly reflecting enhanced cross-talk between storage and executive networks underlying stimulus maintenance. Right frontal alpha and right temporal beta activity correlated positively with memory load during the delay period of a Sternberg-type task using natural syllables (Leiberg et al., 2006b). The alpha increase was consistent with other auditory (Luo et al., 2005; Kaiser et al., 2007a; Kawasaki et al., 2010) and visual short-term memory studies (Sauseng et al., 2005, 2009) and may have reflected increased executive demands and/or the suppression of irrelevant processing.

## Auditory Working Memory for Spatial Sound Features

MEG studies investigating spatial AWM tasks with filtered noise sounds found gamma activity over regions of the putative auditory dorsal space processing stream (Rauschecker and Tian, 2000). When comparing auditory spatial working memory with a non-memory contral task, both maintenance and retrieval of lateralized sounds were accompanied by increased parietal gamma activity (55–70 Hz) (Lutzenberger et al., 2002; Leiberg et al., 2006a). In addition, enhanced frontal gamma activity was found during the final 100 ms of the maintenance period. As in our study with artificial syllables described above (Kaiser et al., 2003), gamma coherence between the putative sensory representation regions and frontal cortex was increased during the delay phase.

Inspired by the hypothesized role of gamma activity for sensory representations (Jensen et al., 2007), we searched for spectral signatures of the short-term maintenance of individual auditory stimuli by contrasting delay-period activations between individual memory stimuli. We performed Fast Fourier Transforms on single trials for about 1.5 Hz-wide frequency bins across the gamma range. The problem of multiple testing was addressed by applying a statistical probability mapping based on permutation tests. When frequency ranges showing significant differences between stimuli were identified, the data were filtered in these frequencies to assess spectral activity time courses.

We identified stimulus-specific components of gamma activity during the maintenance of different sound lateralization angles (Kaiser et al., 2008). Sample stimuli were 200-ms noises convoluted with head-related transfer functions to create virtual lateralization angles of either 15° or 45° with respect to the midsagittal plane. After an 800-ms delay period, these stimuli had to be compared with test stimuli that could either be presented with the same, with a more medial or a more lateral angle. Participants were assigned to two groups who were presented with only right- or left-lateralized stimuli, respectively. For both groups, stimulus-specific gamma activity (55–70 Hz) was found over occipito-parietal cortex contralateral to stimulation. This topography could be considered consistent with the auditory dorsal “where” stream, but might also indicate an involvement of visual spatial imagery. Gamma activity was most pronounced at latencies of 200–500 ms after sound offset, i.e., in the middle of the 800-ms delay phase.

**FIGURE 1:**
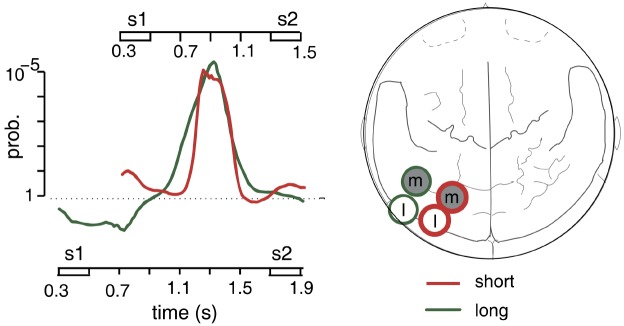
**Stimulus-specific gamma activity to sounds of different lateralization angle in a spatial AWM task.** The graph on the left shows grand-average time courses of a gamma activity differentiation score reflecting the degree to which oscillatory signals differentiate between the two sample stimuli. Positive values indicate a “consistent” differentiation with larger amplitudes to the preferred stimulus, while negative values stand for an “inconsistent” differentiation with larger amplitudes to the non-preferred sound. The amplitude of this difference score was tested against zero to obtain a statistical (*p*-value) time course. Curves were overlaid for both delay durations and aligned for the time point of S2. The red curve (referring to the time axis at the top) shows the short, the green curve (referring to the time axis at the bottom) the long delay period. The map on the right shows the sensor positions showing stimulus-specific effects for the lateral (l) and medial (m) sample sounds during the short (red circles) and the long (green circles) delay durations. Adapted from Kaiser et al. (2009b), copyright 2009 with permission from Elsevier.

This timing of stimulus-specific gamma activity could either have reflected delayed responses to memory sounds or preparatory activations preceding the test stimuli. To decide between these possibilities, a follow-up study used delay durations of either 800 or 1200 ms in separate recording blocks (Kaiser et al., 2009b). The main results of this study are depicted in Figure 1. We replicated stimulus-specific gamma activity (75–100 Hz) over contralateral posterior cortex. For the shorter delay duration, this activity peaked again in the middle of the maintenance phase, i.e., about 400 ms after the offset of the memory stimulus. In contrast, stimulus-specific activity was clearly delayed for the longer delay duration, peaking at around 800 ms after memory stimulus offset. In other words, gamma activity reached its maximum 400 ms before the onset of the test stimulus for both delay durations. The time course of stimulus-specific activity thus seemed to reflect the activation of task-relevant information in preparation for comparison with the test sound.

**FIGURE 2:**
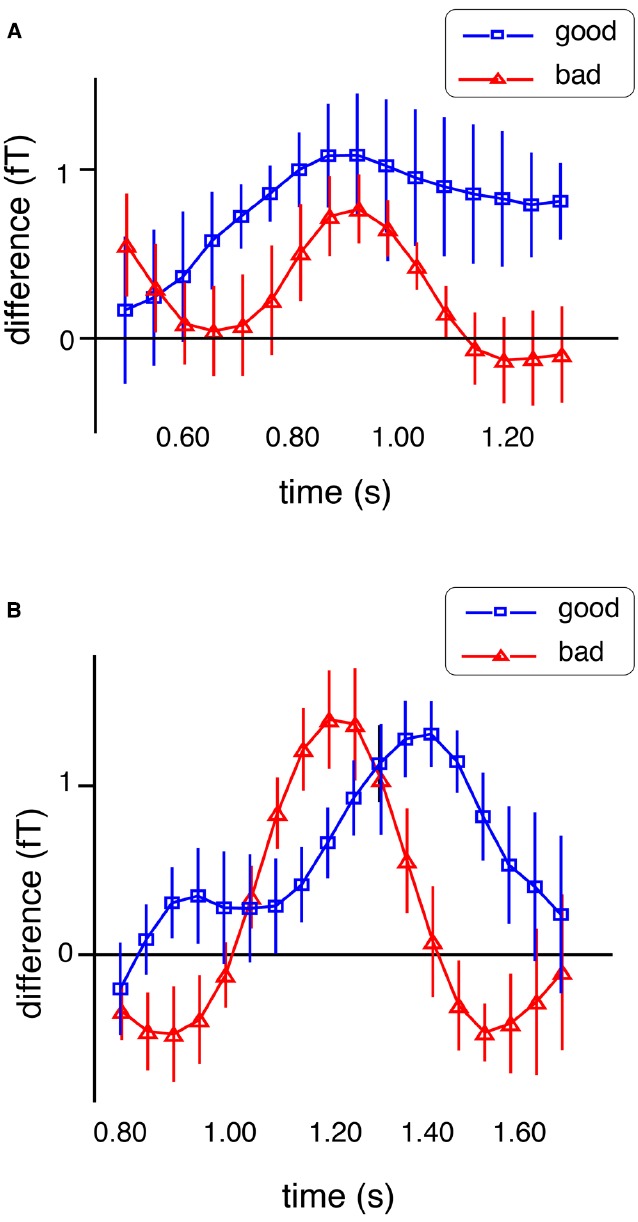
**Time courses of the differentiation score (see legend to Figure 1) for good and bad performers (in blue and red, respectively) for the short delay duration (A) and the long duration (B) in the study by Kaiser et al. (2009b)**.

We also examined the relationship between stimulus-specific gamma activity and task performance. If these signals reflect the activation of task-relevant information, they should predict the accuracy of the comparison with the test stimuli. In both studies (Kaiser et al., 2008, 2009b), we found positive correlations between task performance and gamma activity during the final part of the delay phase. Exploring the nature of this relationship further, we compared gamma activity time courses between better and poorer performers. Interestingly, neither group differed in the absolute magnitude of stimulus-specific activations but in their timing. As shown in Figure 2, better performers showed a more sustained representation of the memorized information until the end of the delay period. Correlations between gamma activity and performance have been reported in a wide variety of paradigms (Rieder et al., 2011). Here they supported the functional relevance of activating representations of the sample sounds for accurate comparisons with the test stimuli.

## Direct Comparisons of Auditory Spatial Versus Non-spatial Working Memory

Studies that compared working memory for sound locations and sound patterns directly supported the notion of dorsal and ventral streams for the processing of auditory spatial and non-spatial information, respectively (Rauschecker and Tian, 2000). In line with this dual-stream model, positive ERP deflections at 300–500 ms after both memory and test stimuli were found at fronto-temporal electrodes for a non-spatial AWM task and at centro-parietal electrodes for a spatial task with 500-ms noise bursts (Alain et al., 2001). Positive maintenance-related ERP shifts during the non-spatial task are at odds with the SAN reported above (e.g., Guimond et al., 2011; Lefebvre et al., 2013). However, several differences between studies make it hard to compare these findings directly: Alain et al. (2001) used longer and spectrally richer sounds and a much shorter delay duration than the studies reporting an SAN (500 versus 2000 ms, respectively), raising the possibility that echoic memory may have been involved rather than short-term memory. Moreover data were shown from a few selected (e.g., fronto-temporal) electrode sites only, whereas the SAN was most pronounced at midline fronto-central sites.

Differences between auditory location and pitch working memory were found also for the N1 component to pure tones serving as test stimuli, suggesting an early onset of segregated processing at about 100 ms (Anourova et al., 2001). The N1 findings were replicated in a subsequent study requiring the memorization of either location or frequency of short sound sequences (Anurova et al., 2003). In addition, sample sounds elicited more negative ERPs at 200 and 400 ms in the frequency than location task and more positive ERPs at 450–650 ms for the location than frequency task. Source analysis of late positive potentials to probe stimuli revealed a predominant involvement of middle temporal cortex in pitch and of occipito-temporal regions in location processing (Anurova et al., 2005). In contrast, a late slow wave was modulated by memory load but did not differ between tasks.

In line with the studies reported above that used simple sounds, an *n*-back working memory task with environmental sounds presented at different virtual locations revealed segregation between spatial and non-spatial processing from about 200 ms onwards in auditory association cortex and fronto-parietal cortex (Alain et al., 2009). In summary, these ERP studies showed an early topographical segregation during encoding and retrieval of spatial versus non-spatial auditory information in accordance with the dual-stream model.

Following up our studies on stimulus-specific gamma activity by comparing non-spatial and spatial AWM directly, we demonstrated the task-dependence of stimulus-specific activations (Kaiser et al., 2009a). The same filtered noise sounds that could differ in frequency and perceived lateralization were used in both tasks. Separate components of gamma activity (50–90 Hz) during the delay phase distinguished between both stimulus features. Different lateralization angles were represented by posterior gamma activity, and different sound frequencies, by fronto-central components. These feature-specific activations peaked at 200–300 ms before the onset of the test stimulus and showed a clear task-dependence: amplitude modulations were observed only when the represented feature was task-relevant. Task performance was correlated both with enhanced activity for the task-relevant stimulus attribute and reduced activity for the task-irrelevant feature. This study showed that representations of auditory features are reactivated depending on task demands and that performance benefits from activating task-relevant and attenuating task-irrelevant representations.

## Summary

The present findings are consistent with the notion of working memory as an emergent property relying on the dynamic interplay between attentional and sensory systems (Pasternak and Greenlee, 2005). EEG and MEG provide measures of neural activity with a sufficiently high temporal resolution to distinguish encoding, maintenance and retrieval in AWM. While there is some evidence for task-specific differences in ERP responses during encoding (Anurova et al., 2003; Lehnert and Zimmer, 2006), most of the studies have focused on the short-term retention of acoustic information. Stimulus maintenance is reflected by sustained ERP deflections whose topography varies with the task-relevant stimulus feature. The maintenance of non-spatial sound attributes like pitch is accompanied by a fronto-central negativity (Guimond et al., 2011). This slow wave reflects variations in memory load and is topographically distinct from more posterior activations during visual working memory (Lefebvre et al., 2013). Source analysis has demonstrated generators in auditory and frontal areas, suggesting that the short-term retention of pitch is partially accomplished by the prolonged activation or the reactivation of the brain regions underlying the perceptual processing of pitch (Grimault et al., 2014). In contrast, sound location seems to be processed by more posterior, parieto-occipito-temporal regions. The topographical differences between sound frequency versus location processing in AWM are consistent with the model of segregated auditory ventral and dorsal streams, respectively (Alain et al., 2001; Kaiser and Lutzenberger, 2003). ERP work comparing individual sound features has demonstrated differential processing of spatial versus non-spatial sound parameters starting from 100 ms after stimulus onset. These differences pertained mainly to encoding, early maintenance and retrieval but were less evident during the later part of a longer retention period (Anurova et al., 2003). Analyses of spectral signals have demonstrated sound feature-specific increases of gamma activity both during maintenance and retrieval. However, representations of task-relevant information were not sustained across the delay period but were temporally related to the onset of the test stimulus (Kaiser et al., 2009b). In contrast, coherence between sensory representation regions and prefrontal cortex showed a sustained increase across the maintenance phases of spatial and non-spatial AWM paradigms (Lutzenberger et al., 2002; Kaiser et al., 2003). In summary, both encoding and retrieval are characterized by the enhanced processing of task-relevant stimuli or stimulus attributes. Maintenance relies on a combination of a prolonged activation or a reactivation of sensory representations and an activation of frontal executive networks with increased coupling between both sets of regions.

While the majority of studies have focused on the maintenance aspect of working memory, research on mental operations on stored sounds is very limited. Working memory operations include the selection of one stored item amongst others, updating the focus of attention or the content of working memory with new items, rehearsal and coping with interference (Bledowski et al., 2010). Shifts of attention to auditory objects held in working memory were associated with the activation of fronto-parietal attention systems, and further temporal and parietal activations distinguished between spatial and category-related attention cues (Backer et al., 2015). Mental transformation and updating of auditory memory contents involved increased frontal and temporal theta power and enhanced fronto-temporal theta phase synchrony (Kawasaki et al., 2010, 2014).

While we have gained substantial knowledge about EEG/MEG signals sensitive to the number of auditory items held in short-term memory, future studies may focus on the neuronal signature coding the precision of individual items (Kumar et al., 2013; Ma et al., 2014). This requires clever experimental designs, sophisticated behavioral analyses and fine-grained analyses of EEG/MEG signals. Furthermore, analyzing connectivity measures in EEG/MEG may help to identify the mechanisms underlying dynamic interactions between the fronto-parietal “working” system that prioritizes, modifies and protects auditory items from interference and the storage system that codes each item representation by a singular activity pattern. These analyses may help to reveal further communalities and differences between visual and auditory working memory.

### Conflict of Interest Statement

The author declares that the research was conducted in the absence of any commercial or financial relationships that could be construed as a potential conflict of interest.

## References

[B1] Alain C. Arnott S. R. Hevenor S. Graham S. Grady C. L. (2001). “What” and “where” in the human auditory system. Proc. Natl. Acad. Sci. U.S.A. 98, 12301–12306. 10.1073/pnas.211209098 11572938 PMC59809

[B2] Alain C. McDonald K. L. Kovacevic N. McIntosh A. R. (2009). Spatiotemporal analysis of auditory “what” and “where” working memory. Cereb. Cortex 19, 305–314. 10.1093/cercor/bhn082 18534993

[B3] Anourova I. Nikouline V. V. Ilmoniemi R. J. Hotta J. Aronen H. J. Carlson S. (2001). Evidence for dissociation of spatial and nonspatial auditory information processing. Neuroimage 14, 1268–1277. 10.1006/nimg.2001.0903 11707083

[B4] Anurova I. Artchakov D. Korvenoja A. Ilmoniemi R. J. Aronen H. J. Carlson S. (2003). Differences between auditory evoked responses recorded during spatial and nonspatial working memory tasks. Neuroimage 20, 1181–1192. 10.1016/S1053-8119(03)00353-7 14568487

[B5] Anurova I. Artchakov D. Korvenoja A. Ilmoniemi R. J. Aronen H. J. Carlson S. (2005). Cortical generators of slow evoked responses elicited by spatial and nonspatial auditory working memory tasks. Clin. Neurophysiol. 116, 1644–1654. 10.1016/j.clinph.2005.02.029 15897006

[B6] Backer K. C. Binns M. A. Alain C. (2015). Neural dynamics underlying attentional orienting to auditory representations in short-term memory. J. Neurosci. 35, 1307–1318. 10.1523/JNEUROSCI.1487-14.2015 25609643 PMC6605545

[B7] Bledowski C. Kaiser J. Rahm B. (2010). Basic operations in working memory: contributions from functional imaging studies. Behav. Brain Res. 214, 172–179. 10.1016/j.bbr.2010.05.041 20678984

[B8] Chao L. L. Knight R. T. (1998). Contribution of human prefrontal cortex to delay performance. J. Cogn. Neurosci. 10, 167–177. 10.1162/089892998562636 9555105

[B9] Drew T. W. McCollough A. W. Vogel E. K. (2006). Event-related potential measures of visual working memory. Clin. EEG Neurosci. 37, 286–291. 10.1177/155005940603700405 17073166

[B10] Gaab N. Gaser C. Zaehle T. Jäncke L. Schlaug G. (2003). Functional anatomy of pitch memory—an fMRI study with sparse temporal sampling. Neuroimage 19, 1417–1426. 10.1016/S1053-8119(03)00224-6 12948699

[B11] Grimault S. Lefebvre C. Vachon F. Peretz I. Zatorre R. Robitaille N. (2009). Load-dependent brain activity related to acoustic short-term memory for pitch: magnetoencephalography and fMRI. Ann. N. Y. Acad. Sci. 1169, 273–277. 10.1111/j.1749-6632.2009.04844.x 19673792

[B12] Grimault S. Nolden S. Lefebvre C. Vachon F. Hyde K. Peretz I. (2014). Brain activity is related to individual differences in the number of items stored in auditory short-term memory for pitch: evidence from magnetoencephalography. Neuroimage 94, 96–106. 10.1016/j.neuroimage.2014.03.020 24642285

[B13] Guimond S. Vachon F. Nolden S. Lefebvre C. Grimault S. Jolicoeur P. (2011). Electrophysiological correlates of the maintenance of the representation of pitch objects in acoustic short-term memory. Psychophysiology 48, 1500–1509. 10.1111/j.1469-8986.2011.01234.x 21824153

[B14] Jensen O. Kaiser J. Lachaux J. P. (2007). Human gamma-frequency oscillations associated with attention and memory. Trends Neurosci. 30, 317–324. 10.1016/j.tins.2007.05.001 17499860

[B15] Jensen O. Mazaheri A. (2010). Shaping functional architecture by oscillatory alpha activity: gating by inhibition. Front. Hum. Neurosci. 4:186. 10.3389/fnhum.2010.00186 21119777 PMC2990626

[B16] Kaiser J. Heidegger T. Wibral M. Altmann C. F. Lutzenberger W. (2007a). Alpha synchronization during auditory spatial short-term memory. Neuroreport 18, 1129–1132. 10.1097/WNR.0b013e32821c553b 17589312

[B17] Kaiser J. Leiberg S. Rust H. Lutzenberger W. (2007b). Prefrontal gamma-band activity distinguishes between sound durations. Brain Res. 1139, 153–162. 10.1016/j.brainres.2006.12.085 17270158

[B18] Kaiser J. Heidegger T. Wibral M. Altmann C. F. Lutzenberger W. (2008). Distinct gamma-band components reflect the short-term memory maintenance of different sound lateralization angles. Cereb. Cortex 18, 2286–2295. 10.1093/cercor/bhm251 18252742 PMC2536701

[B19] Kaiser J. Leiberg S. Lutzenberger W. (2005). Let’s talk together: memory traces revealed by cooperative activation in the cerebral cortex. Int. Rev. Neurobiol. 68, 51–78. 10.1016/S0074-7742(05)68003-8 16443010

[B20] Kaiser J. Lutzenberger W. (2003). Induced gamma-band activity and human brain function. Neuroscientist 9, 475–484. 10.1177/1073858403259137 14678580

[B21] Kaiser J. Lutzenberger W. Decker C. Wibral M. Rahm B. (2009a). Task-and performance-related modulation of domain-specific auditory short-term memory representations in the gamma-band. Neuroimage 46, 1127–1136. 10.1016/j.neuroimage.2009.03.011 19289171

[B22] Kaiser J. Rahm B. Lutzenberger W. (2009b). Temporal dynamics of stimulus-specific gamma-band activity components during auditory short-term memory. Neuroimage 44, 257–264. 10.1016/j.neuroimage.2008.08.018 18790066

[B23] Kaiser J. Ripper B. Birbaumer N. Lutzenberger W. (2003). Dynamics of gamma-band activity in human magnetoencephalogram during auditory pattern working memory. Neuroimage 20, 816–827. 10.1016/S1053-8119(03)00350-1 14568454

[B24] Kawasaki M. Kitajo K. Yamaguchi Y. (2010). Dynamic links between theta executive functions and alpha storage buffers in auditory and visual working memory. Eur. J. Neurosci. 31, 1683–1689. 10.1111/j.1460-9568.2010.07217.x 20525081 PMC2878597

[B25] Kawasaki M. Kitajo K. Yamaguchi Y. (2014). Fronto-parietal and fronto-temporal theta phase synchronization for visual and auditory-verbal working memory. Front. Psychol. 5:200. 10.3389/fpsyg.2014.00200 24672496 PMC3957026

[B26] Klimesch W. Sauseng P. Hanslmayr S. (2007). EEG alpha oscillations: the inhibition-timing hypothesis. Brain Res. Rev. 53, 63–88. 10.1016/j.brainresrev.2006.06.003 16887192

[B27] Koelsch S. Schulze K. Sammler D. Fritz T. Muller K. Gruber O. (2009). Functional architecture of verbal and tonal working memory: an FMRI study. Hum. Brain Mapp. 30, 859–873. 10.1002/hbm.20550 18330870 PMC6871123

[B28] Kumar S. Joseph S. Pearson B. Teki S. Fox Z. V. Griffiths T. D. (2013). Resource allocation and prioritization in auditory working memory. Cogn. Neurosci. 4, 12–20. 10.1080/17588928.2012.716416 23486527 PMC3590753

[B29] Lang W. Starr A. Lang V. Lindinger G. Deecke L. (1992). Cortical DC potential shifts accompanying auditory and visual short- term memory. Electroencephalogr. Clin. Neurophysiol. 82, 285–295. 10.1016/0013-4694(92)90108-T 1372549

[B30] Lefebvre C. Vachon F. Grimault S. Thibault J. Guimond S. Peretz I. (2013). Distinct electrophysiological indices of maintenance in auditory and visual short-term memory. Neuropsychologia 51, 2939–2952. 10.1016/j.neuropsychologia.2013.08.003 23938319

[B31] Lehnert G. Zimmer H. D. (2006). Auditory and visual spatial working memory. Mem. Cogn. 34, 1080–1090 10.3758/BF03193254 17128606

[B32] Leiberg S. Kaiser J. Lutzenberger W. (2006a). Gamma-band activity dissociates between matching and nonmatching stimulus pairs in an auditory delayed matching-to-sample task. Neuroimage 30, 1357–1364. 10.1016/j.neuroimage.2005.11.010 16469508

[B33] Leiberg S. Lutzenberger W. Kaiser J. (2006b). Effects of memory load on cortical oscillatory activity during auditory pattern working memory. Brain Res. 1120, 131–140. 10.1016/j.brainres.2006.08.066 16989782

[B34] Luck S. J. Vogel E. K. (2013). Visual working memory capacity: from psychophysics and neurobiology to individual differences. Trends Cogn. Sci. 17, 391–400. 10.1016/j.tics.2013.06.006 23850263 PMC3729738

[B35] Luo H. Husain F. T. Horwitz B. Poeppel D. (2005). Discrimination and categorization of speech and non-speech sounds in an MEG delayed-match-to-sample study. Neuroimage 28, 59–71. 10.1016/j.neuroimage.2005.05.040 16023868

[B36] Lutzenberger W. Ripper B. Busse L. Birbaumer N. Kaiser J. (2002). Dynamics of gamma-band activity during an audiospatial working memory task in humans. J. Neurosci. 22, 5630–5638. 12097514 10.1523/JNEUROSCI.22-13-05630.2002 PMC6758237

[B37] Ma W. J. Husain M. Bays P. M. (2014). Changing concepts of working memory. Nat. Neurosci. 17, 347–356. 10.1038/nn.3655 24569831 PMC4159388

[B38] Nolden S. Bermudez P. Alunni-Menichini K. Lefebvre C. Grimault S. Jolicoeur P. (2013). Electrophysiological correlates of the retention of tones differing in timbre in auditory short-term memory. Neuropsychologia 51, 2740–2746. 10.1016/j.neuropsychologia.2013.09.010 24036359

[B39] Pasternak T. Greenlee M. W. (2005). Working memory in primate sensory systems. Nat. Rev. Neurosci. 6, 97–107. 10.1038/nrn1603 15654324

[B40] Rauschecker J. P. Tian B. (2000). Mechanisms and streams for processing of “what” and “where” in auditory cortex. Proc. Natl. Acad. Sci. U.S.A. 97, 11800–11806. 10.1073/pnas.97.22.11800 11050212 PMC34352

[B41] Rieder M. K. Rahm B. Williams J. D. Kaiser J. (2011). Human gamma-band activity and behavior. Int. J. Psychophysiol. 79, 39–48. 10.1016/j.ijpsycho.2010.08.010 20828587

[B42] Ruchkin D. S. Berndt R. S. Johnson R. Jr. Ritter W. Grafman J. Canoune H. L. (1997). Modality-specific processing streams in verbal working memory: evidence from spatio-temporal patterns of brain activity. Cogn. Brain Res. 6, 95–113. 10.1016/S0926-6410(97)00021-9 9450603

[B43] Sauseng P. Klimesch W. Heise K. F. Gruber W. R. Holz E. Karim A. A. (2009). Brain oscillatory substrates of visual short-term memory capacity. Curr. Biol. 19, 1846–1852. 10.1016/j.cub.2009.08.062 19913428

[B44] Sauseng P. Klimesch W. Schabus M. Doppelmayr M. (2005). Fronto-parietal EEG coherence in theta and upper alpha reflect central executive functions of working memory. Int. J. Psychophysiol. 57, 97–103. 10.1016/j.ijpsycho.2005.03.018 15967528

